# Chronic Exposure to Continuous Brightness or Darkness Modulates Immune Responses and Ameliorates the Antioxidant Enzyme System in Male Rats

**DOI:** 10.3389/fvets.2021.621188

**Published:** 2021-04-15

**Authors:** Amira Moustafa

**Affiliations:** Department of Physiology, Faculty of Veterinary Medicine, Zagazig University, Zagazig, Egypt

**Keywords:** immunity, circadian rhythm, antioxidant enzymes, cytokines, interleukins

## Abstract

Circadian rhythms are considered vital regulators of immune functions. This study aims to elucidate the effects of chronic circadian disruption on immune functions, clock genes expression, and antioxidant enzymes levels in lymphoid tissues. Adult male Sprague-Dawley rats were subjected to a normal light/dark cycle or either continuous light (LL) or continuous dark (DD) for 8 weeks. The results demonstrated (1) significant decreases in the circulating levels of interleukin 1β, interleukin 6 and tumor necrosis factor alpha (TNF-α) and significant increases in the levels of interleukin 10, interleukin 12, C-reactive protein (CRP) and corticosterone in both LL and DD groups; (2) upregulation in mRNA expression of core clock genes Cry1, Cry2, Per1, Per2, and Per3 in the spleen of the DD group and downregulation in Cry1 and Cry2 genes in the LL group; (3) elevation of total antioxidant capacity (TAC), superoxide dismutase (SOD), catalase (CAT), glutathione peroxidase (GPx), nitric oxide (NO) and the lipid peroxidation marker malondialdehyde (MDA) in the spleen, lymph node and bone marrow of both the LL and DD groups and decreases in the levels of the same markers in the thymus of the LL group; (4) decreased numbers of CD4^+^ and CD8^+^ cells in lymphoid tissues of both the LL and the DD groups; (5) reduced platelets count and suppressed immunoglobulin (IgM, IgE) in the LL and DD groups with marked erythropenia and leukocytosis in the DD group. Taken together, circadian misalignment leads to hematological disruptions, dysregulation of clock genes, and inflammatory mediators, which further enhances the antioxidant enzyme system that is crucial for an organism's adaptation to stresses.

**Graphical Abstract:**
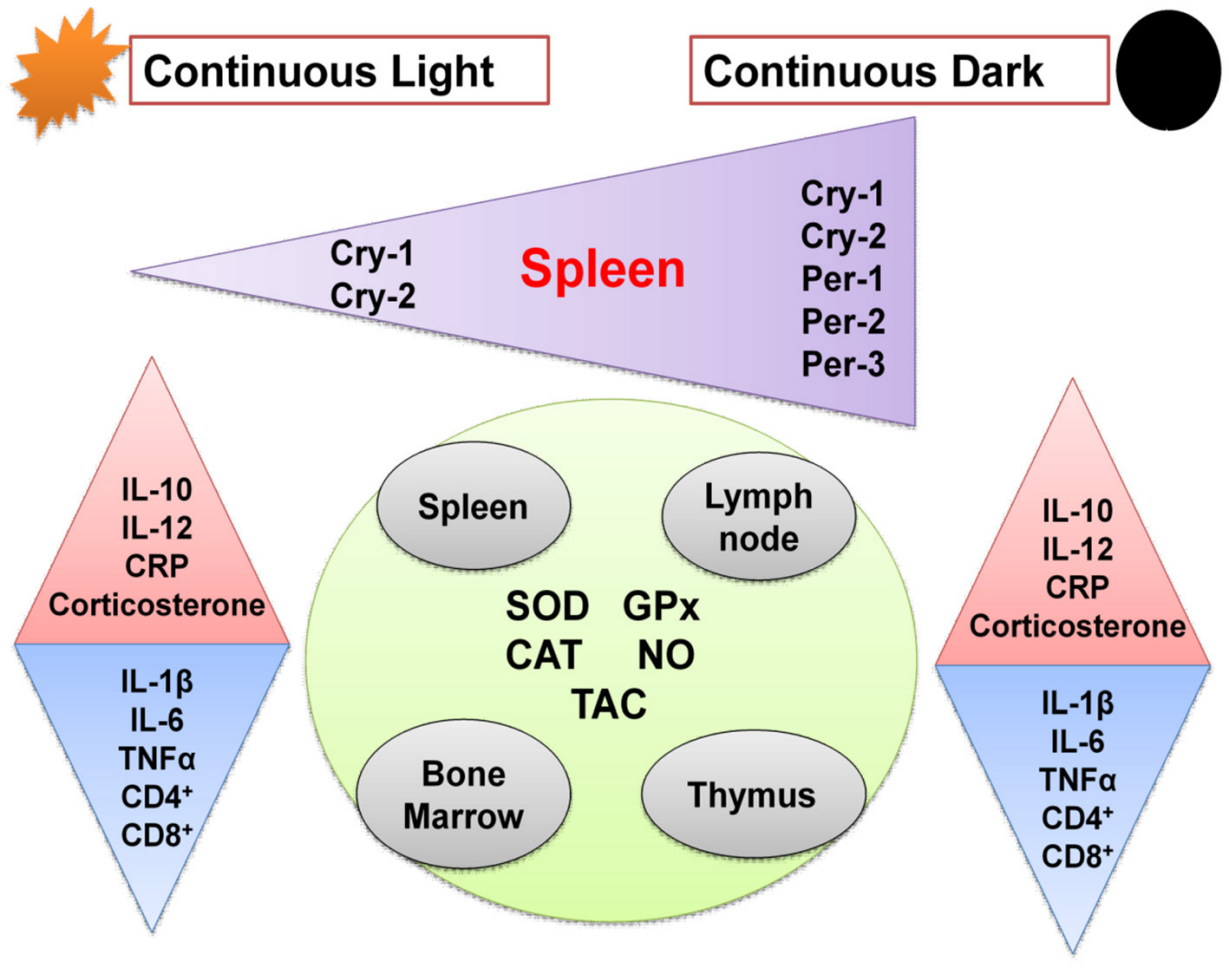
Schematic diagram showing effects of chronic circadian disruption on various immune functions. Regular triangles indicate stimulation and inverted triangles indicate inhibition.

## Introduction

The immune system plays a vital role in maintaining human health by preventing various diseases. It is classified into two types: (1) innate immunity (2) adaptive immunity. Innate immunity is responsible for non-specific but rapid responses, while adaptive immunity mediates specific and long-term protection. In physiology, circadian rhythms are indispensable to life, and abnormalities in circadian rhythms hinder both mental and physical health (1). Accordingly, organisms must modify their biological clocks daily using external cues; the most effective cue is the light stimulus. Circadian rhythms are orchestrated by a master clock located in the hypothalamus. The molecular clockwork involves the circadian Locomotor Output Cycles Kaput (*Clock*) gene, and Aryl hydrocarbon receptor nuclear translocator-like protein 1 (*ARNTL* or *Bmal1*) gene complex that fosters the transcription of clock genes period1–period3 (Per1, Per2, and Per3), cryptochrome-1 (CRY1), cryptochrome-2 (CRY2), and nuclear hormone receptors Rev-erbα (encoded by *Nr1d1*), and Rev-erbβ (encoded by *Nr1d2*). Sequentially, the protein products Per and CRY repress their own transcription, and Rev-erb inhibits BMAL1 transcription (2). The central clock synchronizes several peripheral clocks that virtually exist in all cells, therefore, setting a common rhythm (3). The same molecular components, as in the central clock, are reported to be present in the immune system hematopoietic cell lineages, including macrophages and lymphocytes (4–6), and immune responses are strongly influenced by the major elements of the clock (BMAL1, CLOCK, PERs, CRYs, and REV-ERBs) (Table 1). Also, circadian rhythmicity has been demonstrated in both hormones and cytokines (21). The functions of neutrophils were also shown to be under circadian regulation (22, 23) and may also be essential for normal homeostasis in the absence of inflammation (24). The cellular and humoral components of the immune system in the blood exhibit opposite rhythms. The numbers of hematopoietic stem cells, progenitor cells, and most mature leukocytes, except CD8^+^ T cells (25), peak in the circulation during the resting phase (night for humans and day for rodents) and decreases during the active period (21). Moreover, the proinflammatory cytokine tumor necrosis factor (TNF) and interleukin-1β (IL-1β) peak during the onset of the active phase (21), which may be associated with the circadian onset of diseases. This circadian regulation of immune functions enables organisms to produce their most efficient response when it is most needed and qualifies various functions to be synchronized in time within the immune system and other physiological systems (26).

**Table 1:** Modulation of immune functions by the components of the circadian clock.

**Gene name**	**Protein name**	**Potential Effect**	**References**
*Arntl/ BMAL1*	Aryl hydrocarbon receptor nuclear translocator-like protein 1/ Brain and muscle ARNT-like 1	Proinflammatory	(7, 8)
*Clock*	Circadian locomotor output cycles protein kaput	Proinflammatory	(9, 10)
*CRY1*	Cryptochrome-1	Anti-inflammatory	(11, 12)
*CRY2*	Cryptochrome-2	Anti-inflammatory	(11, 12)
*Nr1d1*	Nuclear receptor subfamily 1 group D member 1/ Rev-erbA-alpha protein	Proinflammatory/ Anti-inflammatory	(13–17)
*Nr1d2*	Nuclear receptor subfamily 1 group D member 2/ Rev-erb-beta protein	Proinflammatory/ Anti-inflammatory	(13–17)
*Per1*	Period circadian protein homolog 1	Anti-inflammatory	(18)
*Per2*	Period circadian protein homolog 2	Proinflammatory	(19)
*Per3*	Period circadian protein homolog 3	Proinflammatory	(20)

When it is dark at night, humans around the world use artificial light (27). Shift work, jet lag, insufficient sleep, and light exposure are usually associated with circadian rhythm disruption (28). Abnormal circadian rhythms have been linked to insomnia (29), obesity (30, 31), and dyslipidemia (31) in humans. Moreover, prolonged exposure to constant light disrupts circadian rhythmic behavior (32–34), causing obesity and type-2 diabetes in rodents (32, 33). The circadian rhythm influences multifaceted immune responses; therefore, in this study, an artificial model of circadian misalignment was conducted by exposure of rats to continuous brightness or darkness for eight consecutive weeks, and the potential effects on the humoral and cellular components of the immune system, clock gene expression, hematological parameters, and antioxidant enzyme activities in different lymphoid tissues, including spleen, thymus, lymph node, and bone marrow were investigated.

## Materials and Methods

### Animals and Experiment Design

All procedures of animal handling were approved by the Ethics Committee of the Faculty of Veterinary Medicine, Zagazig University (*ZU-IACUC/2/F/139/2019*). Adult male Sprague-Dawley rats were purchased from the animal house unit of the Faculty of Veterinary Medicine, Zagazig University. They were housed in standard polycarbonate cages (2 rats per cage) with hardwood chip bedding in temperature-controlled conditions (21–22°C) with free access to water and food *ad libitum*. Rats were adapted to the housing conditions for 2 weeks before starting the actual experiment. Thirty male rats were randomly divided into three groups (10 rats per group): One group was kept under constant light (LL; 24-h light exposure). The other group was kept under constant darkness (DD; 24-h dark exposure). The control group was kept under a cycle of 12 h of light and 12 h of darkness (normal daily cycle) with lights on at 6:00 AM and lights off at 18:00 PM. After 8 weeks of this experiment between 9:00 a.m. to 12:00 p.m., the rats were killed by rapid decapitation without anesthesia to avoid the influence of anesthesia on corticosterone hormone concentrations (35). Immediately following the sacrifice, the trunk blood was collected in BD Vacutainer PST II tubes, allowed to clot, and was subsequently centrifuged at 3,000 g for 20 min for serum separation, which was stored at −20°C until analysis. Spleen, thymus, femur, and axillary lymph nodes were harvested, weighed, and fixed in 10% Neutral buffered formalin for histological and immunohistochemical examinations. Tissue homogenates (spleen, thymus, bone marrow, and axillary lymph nodes) were prepared by homogenization in ice-cold phosphate-buffered saline (10 mg tissue to 100 μl PBS) using a tissue homogenizer, centrifugation at 3,000 rpm for 20 min, and the supernatants were obtained for antioxidant enzymes measurements. For RNA extraction, a piece of the spleen (100 mg) was snap-frozen in liquid nitrogen and stored at −80°C until further processing.

### Hematologic and Biochemical Analysis

Fresh whole blood was collected in tubes containing EDTA, and blood parameters, including red blood cell count (RBCs), platelet count, hemoglobin (Hb) concentration, packed cell volume (PCV), mean corpuscular volume (MCV), mean corpuscular hemoglobin (MCH) and mean corpuscular hemoglobin concentration (MCHC) were examined using a hematological analyzer (Vet Scan HM5, ABAXIS, Hungary). The results were compared to standard reference ranges as indicated previously (36). Plasma total levels of immunoglobulins IgA, IgE, IgG, and IgM were estimated using a rat ELISA kit (Cusabio, San Diego, CA, USA) according to the manufacturer's instructions.

Serum concentrations of interleukins were measured using commercially available ELISA kits: IL-1β (Ray Biotech, Inc. Norcross, GA, USA, with a minimum detectable concentration of 80 pg/mL), IL-6 (Kamiya Biomedical Company, Tukwila, WA, USA, with detection ranges of 1.56–100 pg/mL), IL-10 and IL-12 (Cusabio, San Diego, CA, USA, with detection ranges of 3.12–200 pg/mL). Serum levels of cytokines, including interferon-gamma (IFN-γ) and tumor necrosis factor-alpha (TNF-α), were measured using commercially available ELISA kits (Cusabio, San Diego, CA, USA, with detection ranges of 0.625–40 and 6.25–400 pg/mL, respectively) according to the manufacturer's instructions. The concentration of C-reactive protein (CRP) was quantified using an ELISA kit (MyBioSource, San Diego, CA, USA, with detection ranges of 1.56–100 ng/mL) according to the manufacturer's instructions. Serum corticosterone level was detected using a rat corticosterone ELISA kit (Cusabio, San Diego, CA, USA, with detection ranges of 0.2–40 ng/mL), following the manufacturer's instructions. All samples and standards were run on the same plate. The absorbance was measured at 450 nm on DNM-9602 Microplate Reader (PERLONG, Beijing, China).

### RNA Extraction, cDNA Synthesis, and Quantitative RT-PCR

Total RNA was extracted from the spleen using a GeneJET RNA Purification Kit (GeneJET, Kit, cat no. K0732, Thermo Fisher Scientific Inc., Germany), following the manufacturer's instructions. The yield of total RNA obtained was determined spectrophotometrically at 260 nm. Gene expression was monitored using real-time PCR (StepOne, version 2.1, Applied Biosystems, Foster City, USA). Subsequently, 1 μg of the total RNA from each sample was used for cDNA synthesis followed by PCR amplification cycles using a SensiFAST™ SYBR® Hi-ROX One-Step Kit (Catalog no. PI-50217 V, UK). The thermal cycling profile was 15 min at 45°C for cDNA synthesis followed by 5 min at 95°C for reverse transcriptase inactivation and polymerase activation. PCR amplification consisted of 40 cycles at 95°C for 15 s (DNA denaturation step), 55°C for 20 s (annealing step), and 72°C for 30 s (final extension step). Changes in the expression of each target gene were normalized relative to the mean critical threshold (Ct) values of the β*-actin* housekeeping gene by the ^ΔΔ^Ct method. Primer sequences for the genes are shown in Table 2.

**Table 2:** Sequences of primers used for real-time RT-PCR.

**Gene**	**Forward primer (5′-3′)**	**Reverse primer (5′-3′)**	**Accession number**	**Product size (bp)**
*CRY-1*	AAGGGACTCCGGCTGCACGA	CCCCGGATCACAAACAGGCGA	NM_198750.2	203
*CRY-2*	GCCCAGGAGCCACCAAGCAA	GCATGCACACGCAAACGGCA	NM_133405.2	190
*Per-1*	AGCAGAGGGCGGGTCCAGTT	TTATTGGCCAGGGCGAGCGG	NM_001034125.1	133
*Per-2*	CGCACACGCAACGGGGAGTA	AACGCTGGGGTGCGGAGTCT	NM_031678.1	177
*Per-3*	GAGACGTGCACGGAACCGCA	AGCTATGCTGCTCTGGCCGC	NM_023978.2	121
*β-actin*	TCGTGCGTGACATTAAAGAG	ATTGCCGATAGTGATGACCT	NM_031144	134

### Measurement of Antioxidant Enzymes, Lipid Peroxidation, Total Antioxidant Capacity (TAC), and Nitric Oxide (NO) Levels

Concentrations of the antioxidant enzymes such as catalase (CAT), glutathione peroxidase (GPx), and superoxide dismutase (SOD) in lymphoid tissues, namely, the spleen, thymus, axillary lymph node, and femoral bone marrow, were measured using ELISA kits (MyBioSource, San Diego, CA, USA) according to the manufacturer's instructions. The level of malondialdehyde (MDA), the lipid peroxidation marker, was estimated using commercially available kits (Elabscience Biotechnology Inc., USA) according to the manufacturer's instructions. Total antioxidant capacity (TAC) was measured with a commercially available kit (Cell Biolabs OxiSelect™, San Diego, Inc., CA, USA). The concentrations of nitric oxide (NO) in lymphoid tissues were estimated using an NO assay kit (ELISA; MyBioSource, San Diego, CA, USA) with a detection range of 0.25–200 nmol/L.

### Histomorphological Study

Specimens of the lymphoid tissues, including spleen, thymus, lymph node, and femur bone marrow, were fixed in 10% neutral buffered formalin and further processed for dehydration, clearing, and impregnation using an automatic tissue processor. The specimens were then embedded in paraffin blocks, and serial sections of 3-μm thickness were cut and stained with hematoxylin and eosin. The mounted sections were observed under light microscopy.

### Immunohistochemistry

The procedures of immunohistochemistry were performed using the UltraVision LP Large Volume Detection System (Thermo Fisher Scientific, Fremont, CA, USA) according to the manufacturer's instructions. Briefly, formalin-fixed and paraffin-embedded 4-μm sections from the spleen, thymus, lymph node, and femur bone marrow were deparaffinized, rehydrated, and washed with distilled water. Antigen retrieval was performed by heating for 20 min in citrate buffer (pH 6.0). Endogenous peroxidase activity was inhibited by the UltraVision hydrogen peroxide block for 10 min. Normal goat serum (10%) was used to block the non-specific antibody binding. Automated immunohistochemistry was performed using the Dako Autostainer Model S3400 (Dako Cytomation, Inc., CA). Slides were incubated with CD4 or CD8 primary antibodies (1:50 dilution, Invitrogen; Thermo Fisher Scientific, Inc.) for 1 h at room temperature. The sections were then incubated with horseradish peroxidase polymer for 15 min at room temperature. Finally, the slides were immersed in diaminobenzidine as a chromogen for 10 min. After washing, the slides were counterstained with Mayer's hematoxylin. Negative control slides were prepared by incubation in phosphate-buffered saline instead of the primary antibody. The sections were examined for the presence of CD4^+^ and CD8^+^ T-lymphocytes. For each CD4- and CD8-stained section, eight random fields with a minimum of 100 cells each were counted at a magnification of 40× on a computer screen connected to a microscope. This method of counting was effective for the spleen, thymus, and lymph node sections but not for bone marrow due to the spongy nature of bone marrow. Therefore, a semi-quantitative analysis of bone marrow images was performed using ImageJ Fiji software version 2.1 (37).

### Statistics

Data are presented as mean ±SEM. One-way ANOVA was used to determine the significant differences between groups, and Student's *t*-test was used to assess the differences between two independent groups. *P* < 0.05 was considered statistically significant.

## Results

### Effect of Circadian Rhythms Alterations on Hematological Parameters

There are three types of blood cells with distinct functions: erythrocytes (RBCs), which are responsible for transporting oxygen from the lung to tissues; leukocytes, which coordinate and implement the immune responses; and thrombocytes (platelets), which are vital for hemostasis. Table 3 compares the different hematological parameters between control rats and those exposed to either continuous light or dark. Constant brightness significantly decreased platelet count, Hb concentrations, MCH, and MCHC with no effect on RBC count or PCV values. However, constant darkness significantly decreased all the examined hematological parameters including platelet count, RBC count, Hb concentrations, PCV, and MCHC while increasing values of MCV and MCH.

**Table 3:** Comparison of hematological parameters in different groups.

	**Control**	**Light (LL)**	**Dark (DD)**	**ANOVA F value**
				**(df = 2, 21)**
RBCs (×10^6^/cmm)	5.728 ± 0.347^a^	5.175 ± 0.196^a^	4.122 ± 0.055^b^	18.458 *P <* 0.001
Platelet count (×10^3^/cmm)	903 ± 1.816^a^	736.75 ± 3.137^b^	666.75 ± 5.687^b^	13.031 *P <* 0.001
HB/dl	18.375 ± 0.572^a^	15.25 ± 0.191^b^	14.737 ± 0.139^b^	15.183 *P <* 0.001
PCV (%)	47.872 ± 0.519^a^	45.702 ± 0.259^a^	42.206 ± 0.117^b^	12.457 *P <* 0.001
MCV (fl)	85.312 ± 0.792^a^	89.455 ± 0.637^a^	101.707 ± 0.359^b^	17.346 *P <* 0.001
MCH (pg)	31.87 ± 0.394^a^	29.52 ± 0.322^b^	35.495 ± 0.071^b^	28.014 *P <* 0.001
MCHC (g/dl)	37.52 ± 0.358^a^	33.383 ± 0.168^b^	34.945 ± 0.247^b^	13.439 *P <* 0.001

*Data are presented as Mean ± SEM (n = 10/group). Values with the same superscript letters in the given row are not significantly different, whereas those with different superscript letters are significantly different as judged by Student's t-test. Statistical differences between groups were estimated by ANOVA, df: the degree of freedom. RBCs, Red blood cells; HB, Hemoglobin; PCV, Packed cell volume; MCV, Mean Corpuscular Volume; MCH, Mean Corpuscular Hemoglobin and MCHC, Mean Corpuscular Hemoglobin Concentration*.

The effects of different light/dark regimes on total and differential leukocyte counts are summarized in Table 4. Continuous light exposure incited basophilia, eosinophilia, and monocytosis. However, continuous darkness induced significant leukocytosis, monocytosis, and neutrophilia along with lymphopenia.

**Table 4:** Changes in total and differential counts of leucocytes in different groups.

	**Control**	**Light (LL)**	**Dark (DD)**	**ANOVA F value**
				**(df = 2, 21)**
WBCs (×10^3^/cmm)	7.388 ± 0.279^a^	6.458 ± 0.535^a^	10.128 ± 0.555^b^	13.829 *P <* 0.001
Lymphocytes (%)	84.27 ± 0.106^a^	84.565 ± 0.192^a^	80.433 ± 0.224^b^	15.866 *P <* 0.001
absolute value	6.23 ± 0.218	5.460 ± 0.426	8.15 ± 0.51	
Neutrophils (%)	12.25 ± 0.529^a^	10.75 ± 0.716^a^	16 ± 0.52^b^	11.927 *P <* 0.001
absolute value	905 ± 0.055	0.694 ± 0.065	1.62 ± 0.18	
Basophils (%)	0.27 ± 0.069^a^	0.333 ± 0.06^b^	0.228 ± 0.208^ac^	8.857 *P <* 0.001
absolute value	0.0199 ± 0.0007	0.0215 ± 0.001	0.023 ± 0.001	
Eosinophils (%)	1.06 ± 0.099^a^	1.266 ± 0.06^b^	0.988 ± 0.082^ac^	22.945 *P <* 0.001
absolute value	0.078 ± 0.002	0.0818 ± 0.007	0.1001 ± 0.007	
Monocytes (%)	1.48 ± 0.118^a^	2.232 ± 0.134^b^	1.616 ± 0.057^c^	58.824 *P <* 0.001
absolute value	0.109 ± 0.006	0.144 ± 0.009	0.163 ± 0.012	

*Data are expressed as Mean ± SEM (n = 10/group). Values with the same superscript letters in the given row are not significantly different, whereas those with different superscript letters are significantly different as judged by Student's t-test. Statistical differences between groups were estimated by ANOVA, df, the degree of freedom; WBCs, White blood cells*.

The changes in plasma immunoglobulin concentrations following circadian disruption are illustrated in Table 5. Both IgE and IgG were significantly lowered by constant brightness. However, constant darkness significantly decreased the levels of IgE, IgG, and IgM, and sharply increased the level of IgA.

**Table 5:** Changes in serum levels of immunoglobulins in different groups.

**Immunoglobulin (ng/ml)**	**Control**	**Light (LL)**	**Dark (DD)**	**ANOVA F value**
				**(df = 2, 21)**
IgA	592.5 ± 60.407^a^	981.25 ± 174.21^a^	1268.75 ± 98.623^b^	4.672 *P <* 0.05
IgE	8.775 ± 1.228^a^	5.43125 ± 0.295^b^	5.29375 ± 0.278^b^	6.968 *P <* 0.01
IgG	678.75 ± 38.146^a^	510.625 ± 47.117^b^	337.5 ± 29.565^c^	19.199 *P <* 0.001
IgM	376.25 ± 23.580^a^	292.125 ± 60.517^ac^	145.625 ± 21.597^b^	8.722 *P <* 0.01

*Data are presented as Mean ± SEM (n = 10/group). Values with the same superscript letters in the given row are not significantly different, whereas those with different superscript letters are significantly different as judged by Student's t-test. Statistical differences between groups were estimated by ANOVA, df, the degree of freedom*.

### Effect of Circadian Rhythms Alterations on Cytokines, Corticosterone, and CRP Concentrations

**Figure 1:**
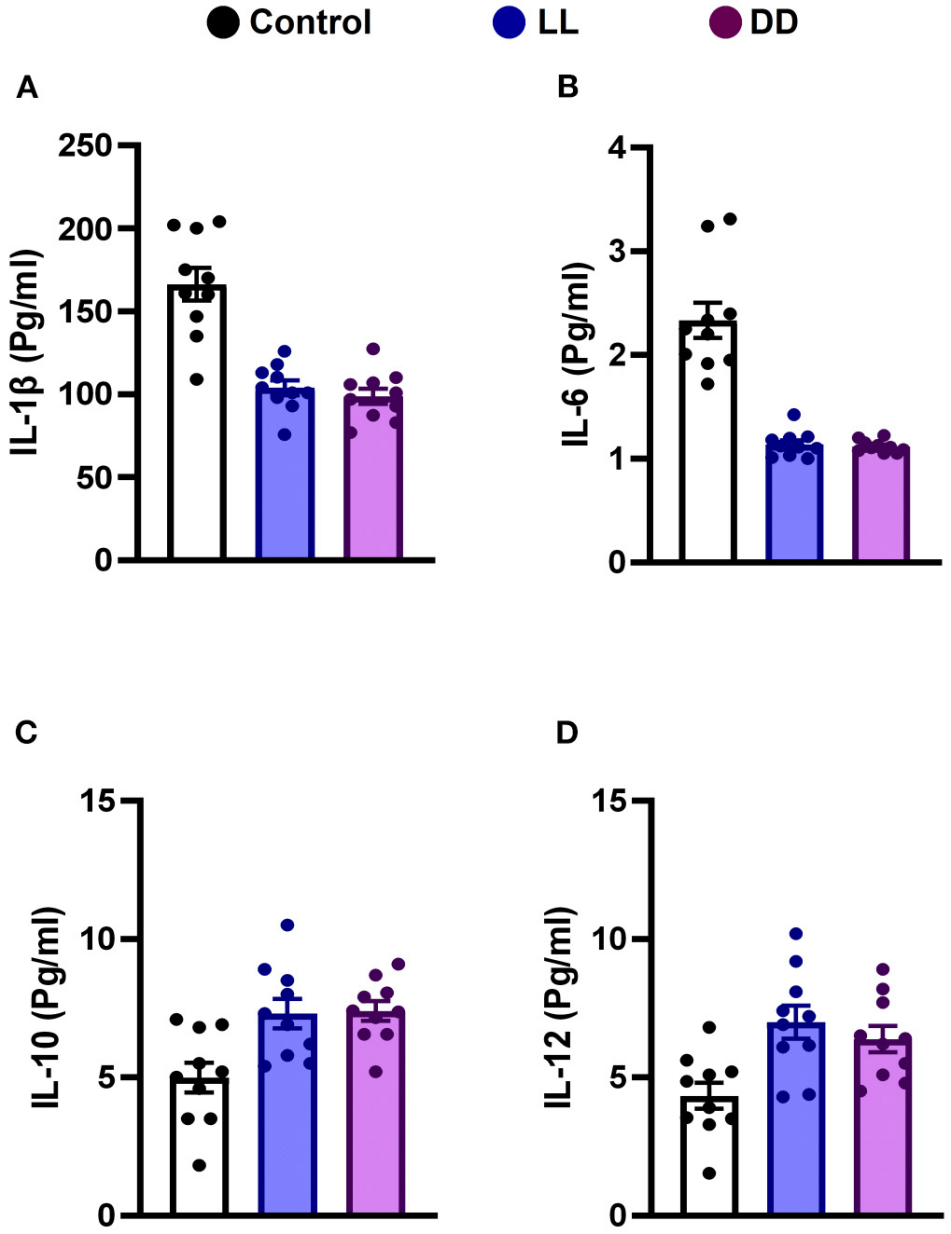
Effect of changing dark/light cycle on different interleukins (pg/ml) in male rats. Serum levels of **(A)** IL-1β, **(B)** IL-6, **(C)** IL-10, and **(D)** IL-12 in rats exposed to continuous light (LL), continuous darkness (DD), or normal daily cycle. Data are presented as means ± SEM (*n* = 10/group).

**Figure 2:**
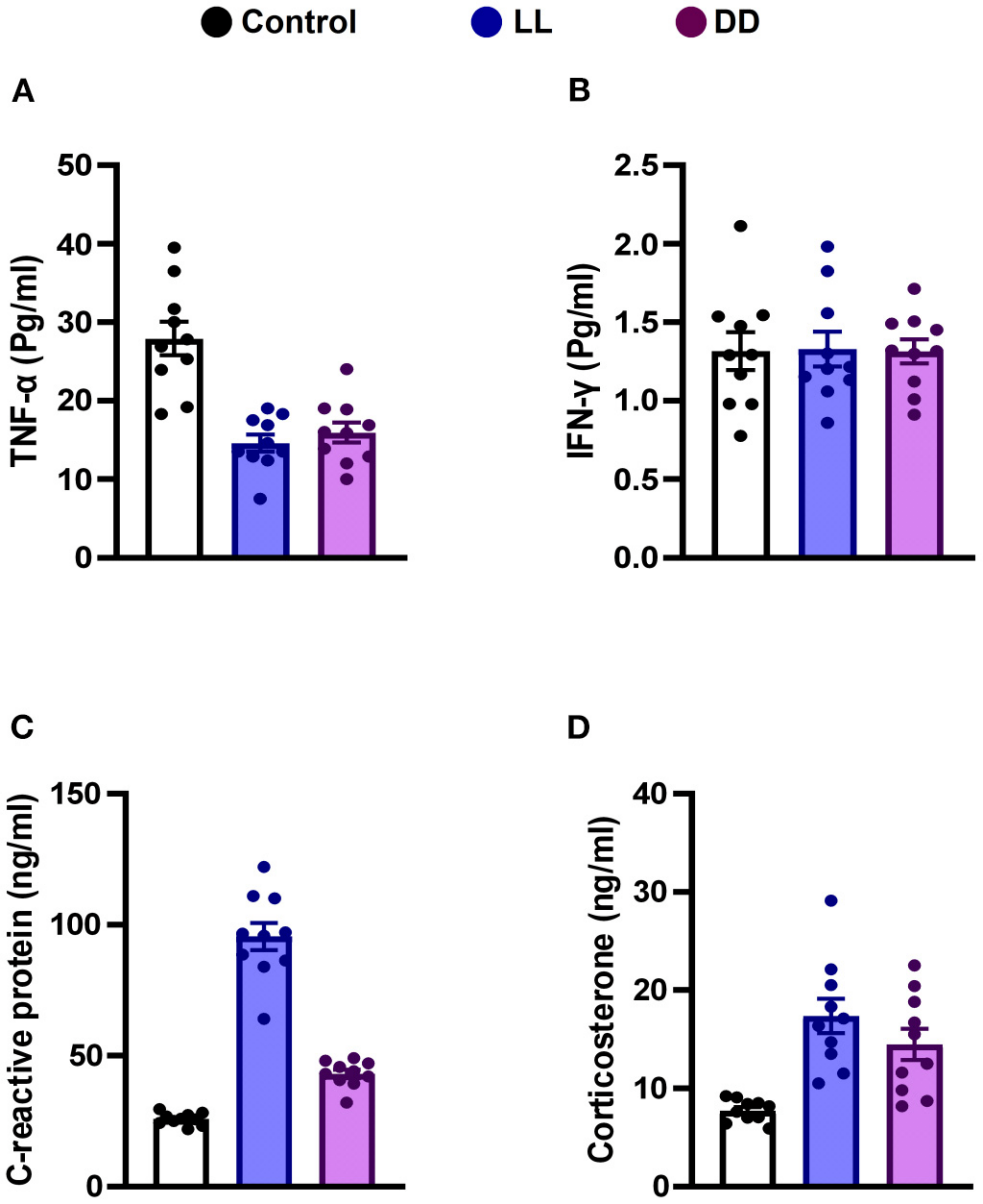
Effect of changing dark/light cycle on the levels of cytokines in male rats. Serum levels (Pg/ml) of **(A)** interferon-gamma (INF-γ), and **(B)** tumor necrosis factor-alpha (TNF-α). **(C)** plasma levels (ng/ml) of C-reactive protein (CRP). **(D)** corticosterone concentrations (ng/ml) in rats exposed to continuous light (LL), continuous darkness (DD) or normal daily cycle. Values are presented as means ± SEM (*n* = 10/group).

Circulating levels of proinflammatory cytokines (IL-1β, IL-6, TNF-α, and IFN-γ), anti-inflammatory cytokine (IL-10), the immunoregulatory cytokine (IL-12), corticosterone, and the marker of inflammation, CRP, were measured following different light/dark exposure protocols. Both constant light exposure and constant dark exposure significantly decreased the circulating levels of IL-1β (Figure 1A; *P* < 0.05 by Student's *t*-test), IL-6 (Figure 1B; *P* < 0.001 by Student's *t*-test), and TNF-α (Figure 2A; *P* < 0.01 and *P* < 0.05 by Student's *t*-test, respectively) while significantly increasing the levels of IL-10 (Figure 1C; *P* < 0.05 and *P* < 0.01, respectively, by Student's *t*-test) and IL-12 (Figure 1D; *P* < 0.01 and *P* < 0.05 by Student's *t*-test, respectively) with no change in the level of IFN-γ (Figure 2B). Levels of CRP were sharply elevated by continuous light and moderately elevated by continuous dark exposure (Figure 2C; *P* < 0.001 by Student's *t*-test). Both continuous light and continuous dark exposure significantly increased the serum concentrations of corticosterone (Figure 2D; *P* < 0.001 and *P* < 0.01 by Student's *t*-test, respectively).

### Effect of Circadian Rhythms Alterations on Clock Genes Expression in Rat Spleen

**Figure 3:**
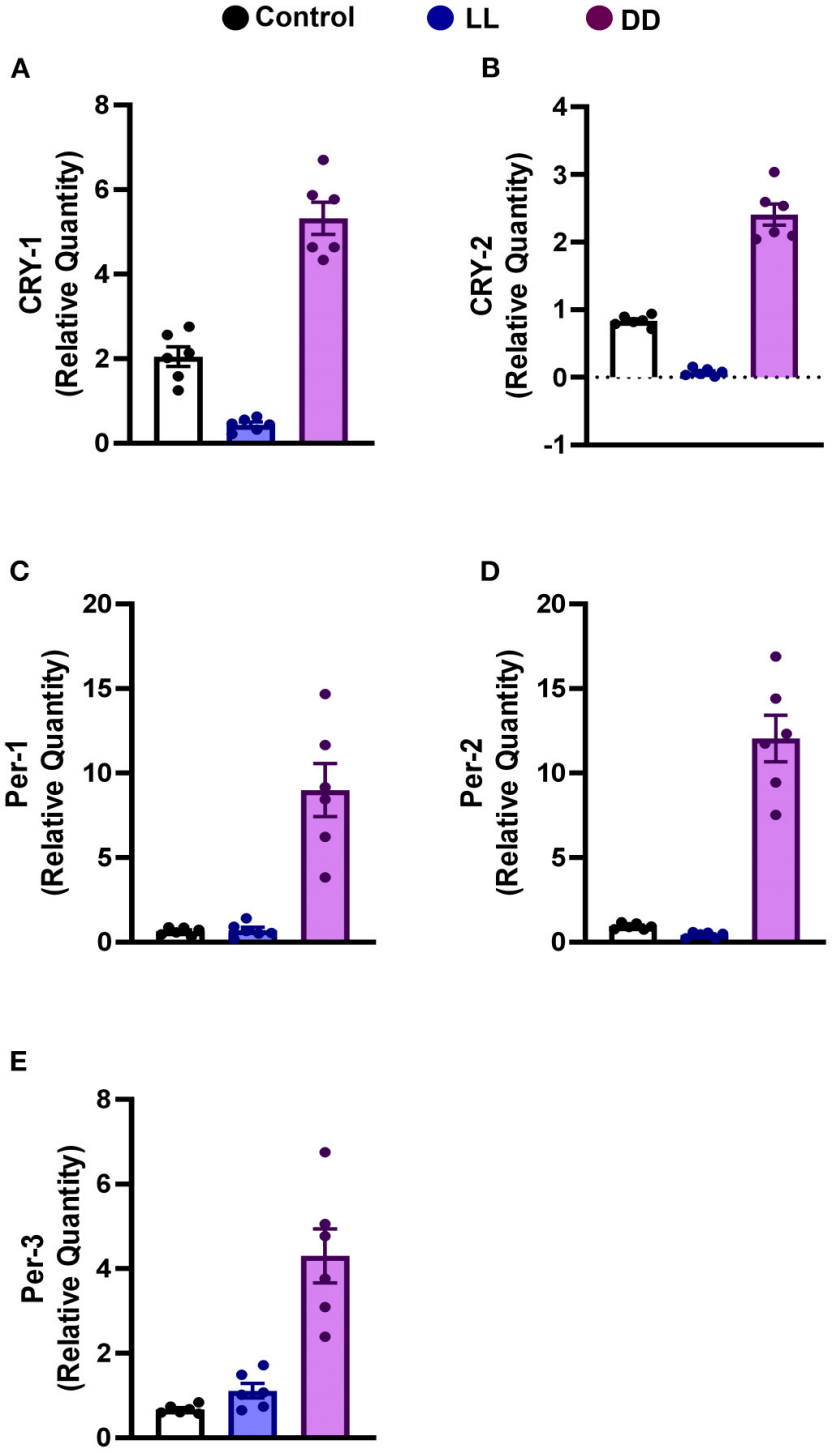
Effect of changing dark/light cycle on mRNA levels of clock genes in the rat spleen. Relative expression of mRNA of **(A)** Cry-1, **(B)** Cry-2, **(C)** Per-1, **(D)** Per-2, and **(E)** Per-3 in rat spleen exposed to continuous light (LL), continuous darkness (DD), or normal daily cycle. Each value is the mean ± SEM.

Constant brightness significantly decreased the expression level of *CRY1* and *CRY2* mRNA (Figures 3A,B, respectively; *P* < 0.05 and *P* < 0.001 by Student's *t*-test, respectively), whereas the other clock genes, including *Per1, Per2*, and *Per3*, remained unchanged (Figures 3C–E, respectively). In contrast, constant darkness significantly increased the mRNA expression of core clock genes, namely *CRY1* (Figure 3A; *P* < 0.01 by Student's *t*-test), *CRY2, Per1* (Figures 3B,C; *P* < 0.05 by Student's *t*-test), *Per2* (Figure 3D; *P* < 0.01 by Student's *t*-test), and *Per3* (Figure 3E; *P* < 0.05 by Student's *t*-test).

### Effect of Circadian Rhythms Alterations on the Levels of Antioxidant Enzymes, Total Antioxidant Capacity, Lipid Peroxidation, and NO Production in Different Lymphoid Organs

**Figure 4:**
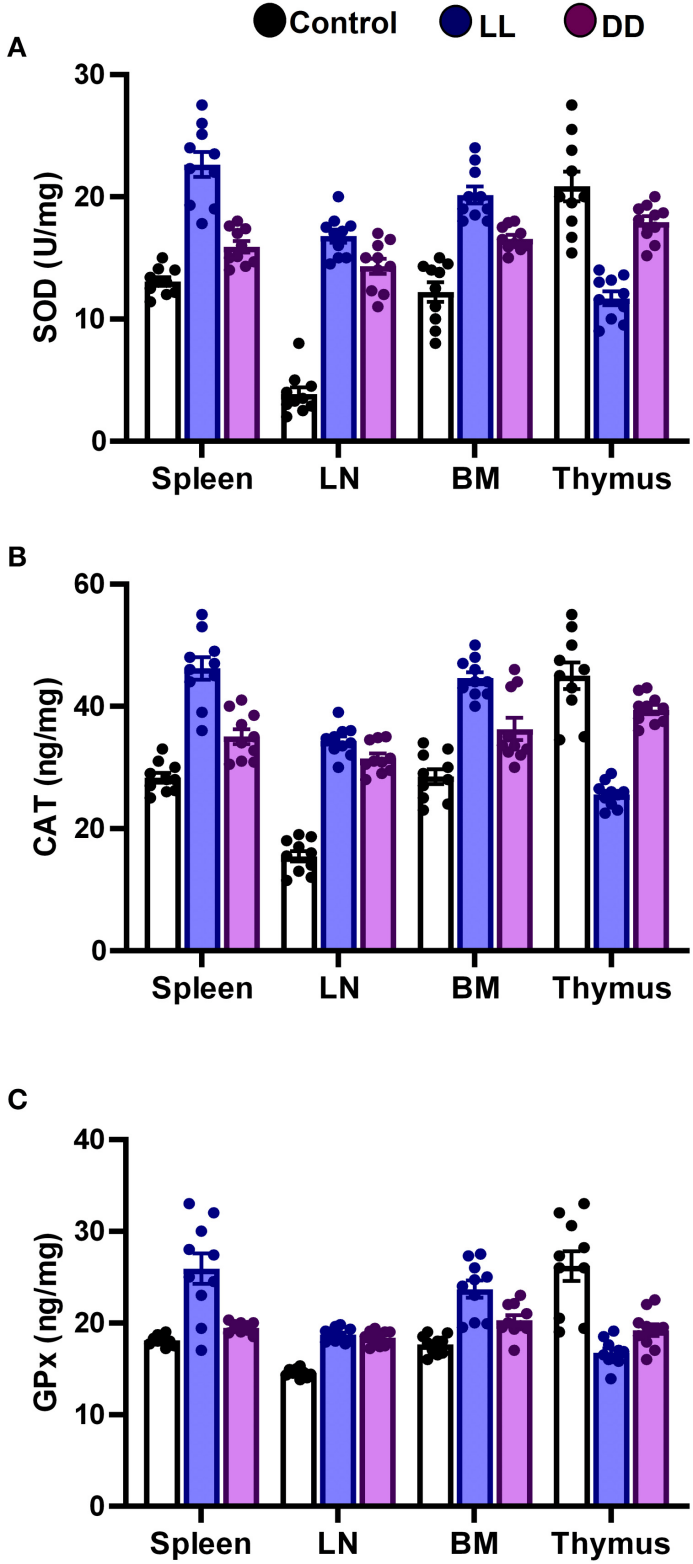
Effect of changing dark/light cycle on antioxidant enzymes levels in different lymphoid organs. Levels of **(A)** superoxide dismutase (SOD, U/ml), **(B)** catalase (CAT, ng/ml), and **(C)** glutathione peroxidase (GPx, ng/ml) in spleen, lymph node, bone marrow, and thymus of male rats after exposure to continuous light (LL), continuous darkness (DD) or normal daily cycle. Values are expressed as means ± SEM (*n* = 10/group).

**Figure 5:**
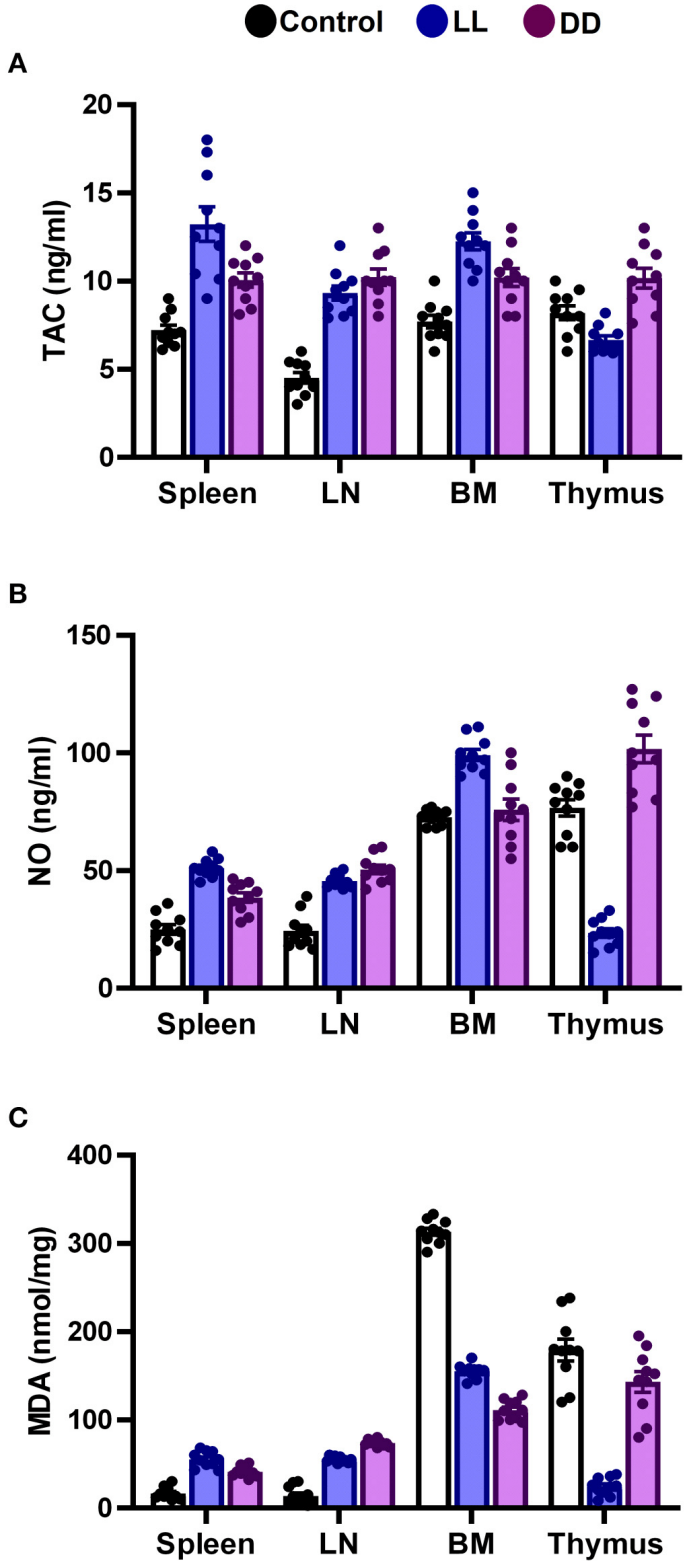
Effect of changing dark/light cycle on total antioxidant capacity, nitric oxide production, and lipid peroxidation in different lymphoid organs. **(A)** Total antioxidant capacity (TAC, ng/ml), **(B)** nitric oxide (NO, nmol/ml), and **(C)** malondialdehyde (MDA, nmol/mg) in the spleen, lymph node, bone marrow, and thymus of male rats exposed to continuous light (LL), continuous darkness (DD) or normal daily cycle. Data are presented as means ± SEM (*n* = 10/group).

In the spleen, both continuous light and continuous dark exposure significantly increased the concentrations of SOD, CAT, and GPx and also increased TAC (Figures 4A–C; Figure 5A, respectively; *P* < 0.001 by Student's *t*-test). Significant elevations in NO production (Figure 5B; *P* < 0.001 by Student's *t*-test) and in the lipid peroxidation biomarker MDA (Figure 5C; *P* < 0.001 by Student's *t*-test) were also demonstrated.

In the thymus, continuous brightness significantly reduced the levels of all examined antioxidant enzymes including SOD, CAT, and GPx (Figures 4A, 5C; *P* < 0.001 by Student's *t*-test), and hence, TAC was also decreased (Figure 5A; *P* < 0.001 by Student's *t*-test). Production of NO and MDA was significantly diminished (Figures 5B,C, respectively; *P* < 0.001 by Student's *t*-test). Continuous darkness showed no effects on the levels of SOD, CAT, and MDA but significantly decreased the level of GPx (Figure 4C; *P* < 0.01 by Student's *t*-test). However, TAC was significantly increased (Figure 5A; *P* < 0.001 by Student's *t*-test), and NO production was elevated as well (Figure 5B; *P* < 0.05 by Student's *t*-test).

In the lymph node, both constant brightness and constant darkness significantly increased all the examined parameters including SOD, CAT, GPx, TAC, MDA, and NO production (Figures 4A–C; Figures 5A–C, respectively; *P* < 0.001 by Student's *t*-test).

In the bone marrow, continuous light and continuous dark exposure significantly increased the levels of SOD, CAT, and GPx (Figures 4A–C, respectively; *P* < 0.001 by Student's *t*-test), and as a consequence, TAC was significantly increased as well (Figure 5A; *P* < 0.001 by Student's *t*-test). However, production of NO was elevated only by constant light exposure (Figure 5B; *P* < 0.001 by Student's *t*-test), while remaining unchanged by constant darkness. The lipid peroxidation marker, MDA, was greatly inhibited by both continuous brightness and darkness (Figure 5C; *P* < 0.001 by Student's *t*-test).

### Effect of Circadian Rhythms Alterations on the Immunohistochemical Expression of CD4+ and CD8+ Cells in Different Lymphoid Organs

**Figure 6:**
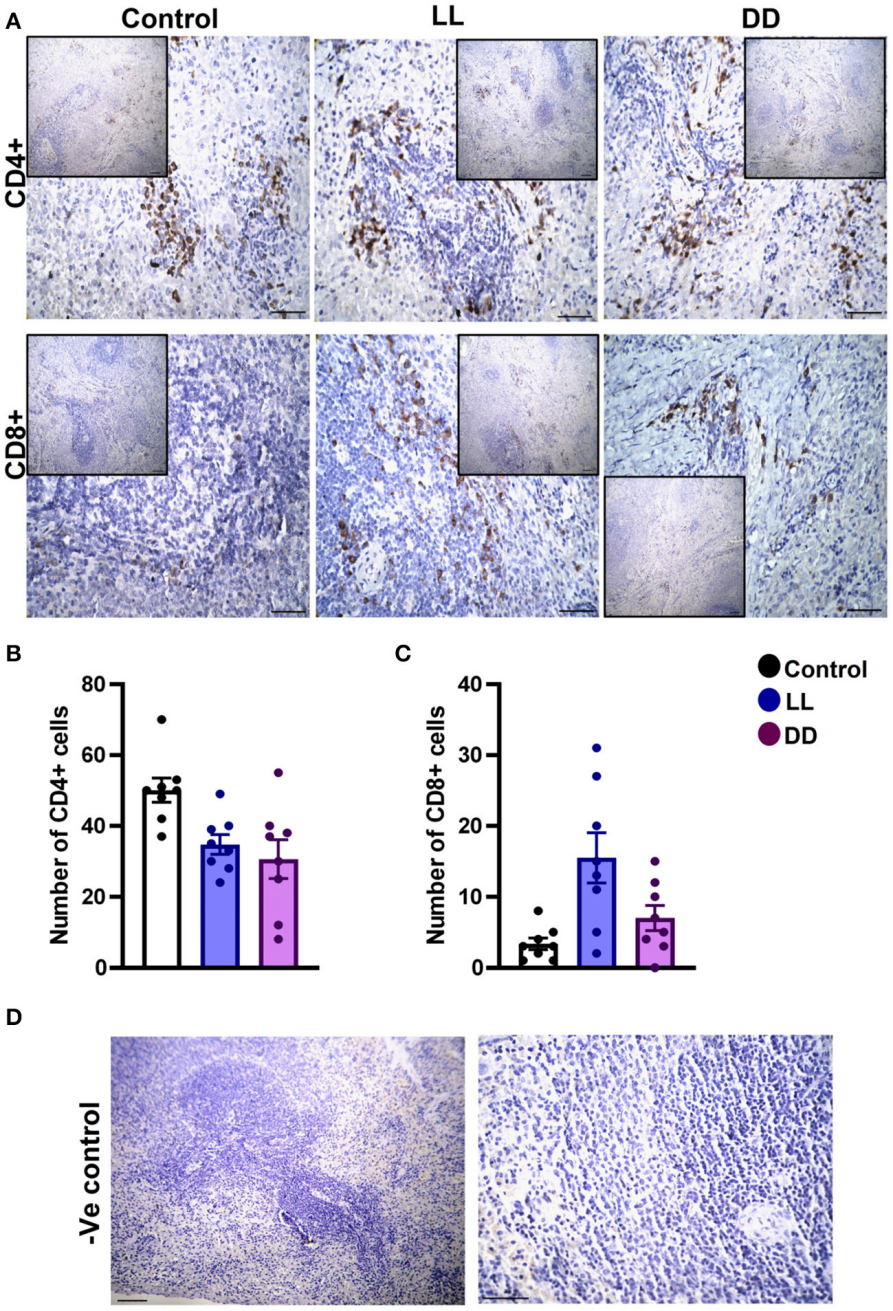
Immunohistochemistry expression of CD4 and CD8 in rat spleen. **(A)** Immunohistochemical detection of CD4^+^ and CD8^+^ cells in rat spleen, respectively. Cells stained positively by the antibody are brown, cell nuclei are counterstained with hematoxylin (blue). **(B,C)** Graphs showing numbers of CD4^+^ and CD8^+^ cells, respectively, in the spleen of control, continuous light (LL) and continuous dark (DD)-exposed rats. Values are expressed as means ± SEM. The count was done in 8 randomly selected sections in each group with 40 × magnifications. **(D)** Representative images of spleen negative control slides (without primary antibody). Scale bar, 50 μm.

**Figure 7:**
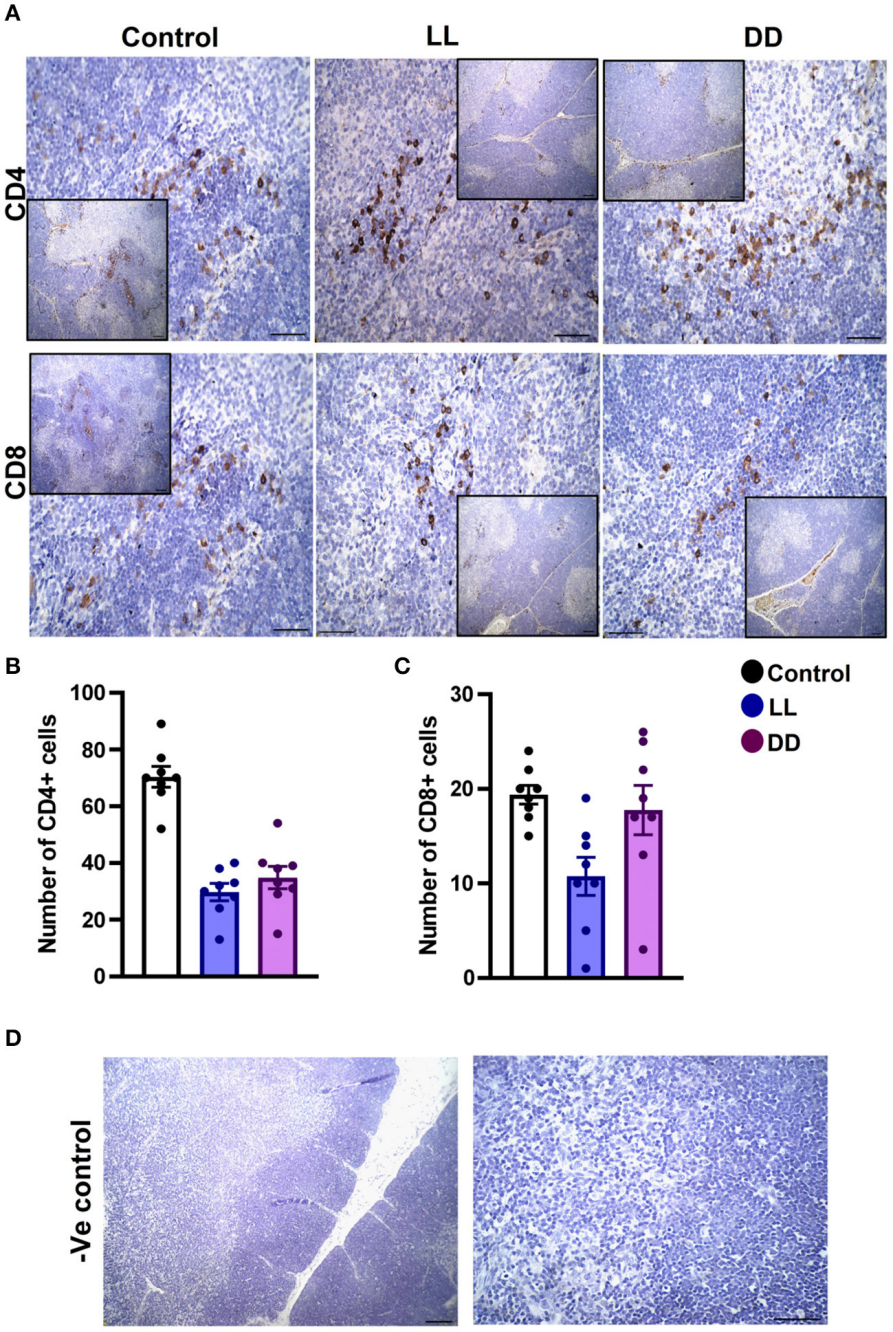
Immunohistochemistry expression of CD4 and CD8 in rat thymus. **(A)** Representative photomicrographs showing the immunohistochemical detection of CD4^+^ and CD8^+^ cells in rat thymus. Positive cells are stained in brown, while cell nuclei are counterstained with hematoxylin (blue). **(B,C)** Graphs showing the mean numbers (± SEM) of CD4^+^ and CD8^+^ cells in the thymus of male rats subjected to continuous brightness (LL), continuous darkness (DD), or normal daily cycle. **(D)** Representative photomicrographs demonstrating negative control slides of the thymus (no primary antibody). Scale bar, 50 μm.

In the spleen, CD4^+^ cells were noticed mainly in the white pulp with some localization in the red pulp (Figure 6A). The number of CD4^+^ cells was significantly decreased by continuous light and continuous dark exposure when compared to control (Figure 6B; *P* < 0.01 by Student's *t*-test). The expression of CD8^+^ cells was faint in the control group (Figure 6A), and the number of CD8^+^ cells was markedly increased by continuous light exposure (Figure 6C; *P* < 0.01 by Student's *t*-test). The thymuses of the control group showed a strong expression of CD4^+^ cells, which was significantly diminished by constant brightness and constant darkness (Figures 7A,B, respectively; *P* < 0.001 by Student's *t*-test). The count of CD8^+^ cells was significantly decreased by constant light exposure (Figure 7C; *P* < 0.01 by Student's *t*-test), while the expression revealed no change after constant-dark exposure.

**Figure 8:**
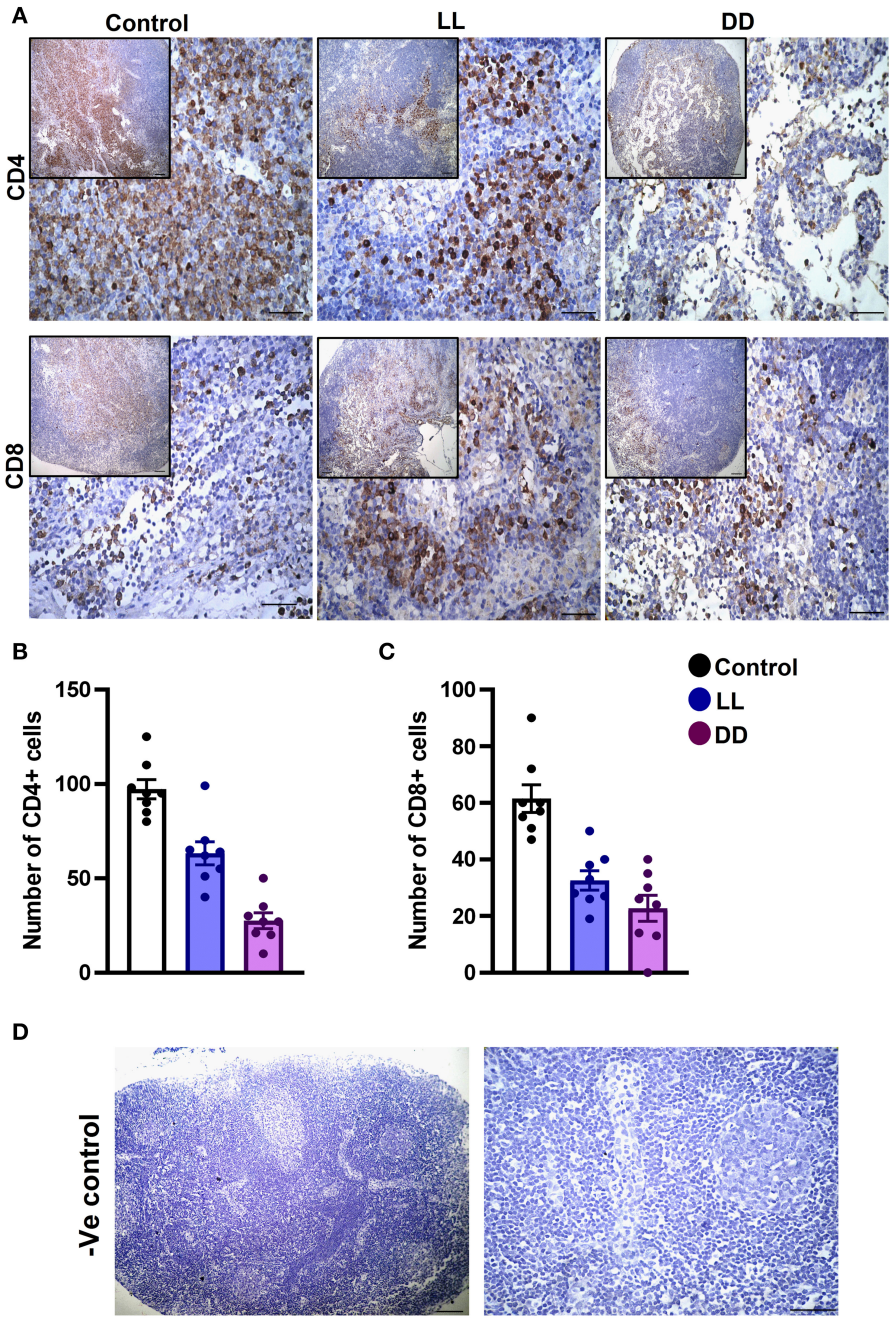
Immunohistochemistry expression of CD4 and CD8 in the rat lymph node. **(A)** Representative images demonstrating the immunohistochemical expression of CD4^+^ and CD8^+^ in the lymph node of rats exposed to different light regimes. **(B,C)** Count of CD4^+^ and CD8^+^ cells in 8 randomly selected sections of the lymph node in each group with 40× magnification. Data are presented as means ± SEM. **(D)** Photomicrographs showing negative control sections of lymph node (without primary antibody). Scale bar, 50 μm.

**Figure 9:**
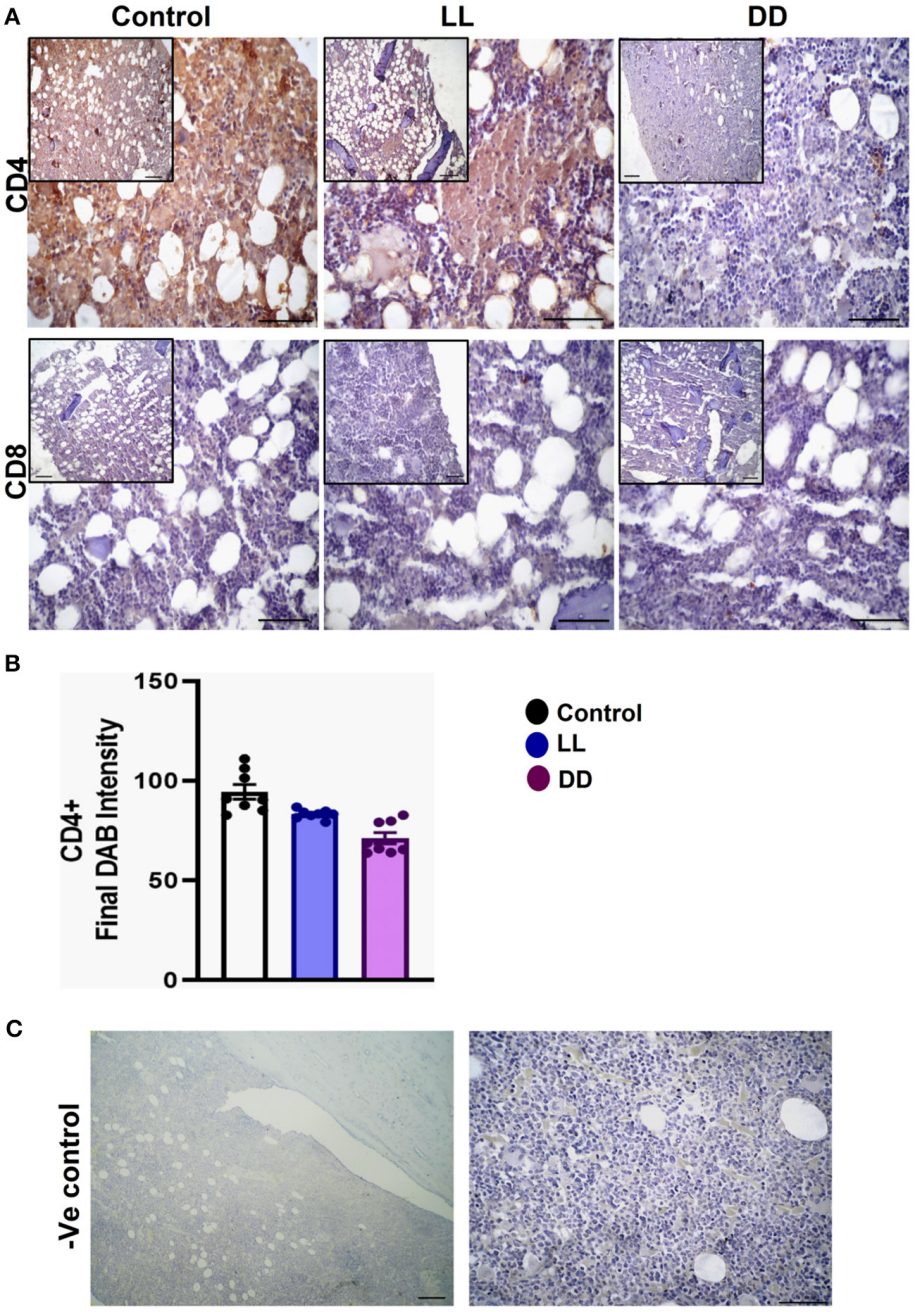
Immunohistochemistry expression of CD4 and CD8 in the bone marrow. **(A)** Representative images showing the immunohistochemical expression of CD4^+^ and CD8^+^ in rat bone marrow (near epiphysis) exposed to a normal daily cycle, continuous brightness (LL), or continuous darkness (DD). **(B)** CD4^+^ final DAB intensity in bone marrow sections of the control, LL, and DD groups. Data are expressed as means ± SEM. **(C)** Negative control sections of bone marrow with no primary antibody. Scale bar, 50 μm.

The axillary lymph nodes of the control group demonstrated a high number of CD4^+^ cells, which significantly decreased with constant light or constant-dark exposure (Figures 8A,B, respectively; *P* < 0.001 by Student's *t*-test). The number of CD8^+^ cells significantly declined with continuous light exposure or continuous dark exposure (Figures 8A,C, respectively; *P* < 0.001 by Student's *t*-test). In the bone marrow, strong expression of CD4^+^ cells was noticed in the stroma of the control group, which declined with either continuous light or continuous dark exposure as concluded by the semi-quantitative analysis of the representative images (Figure 9B, *P* < 0.05 and P < 0.001 by Student's *t*-test, respectively). However, no obvious expression of CD8^+^ cells was noticed in any of the groups (Figure 9A). Therefore, the semi-quantitative image analysis for CD8 expression was not performed.

### Effect of Circadian Rhythms Alterations on the Morphology of Different Lymphoid Organs

**Figure 10:**
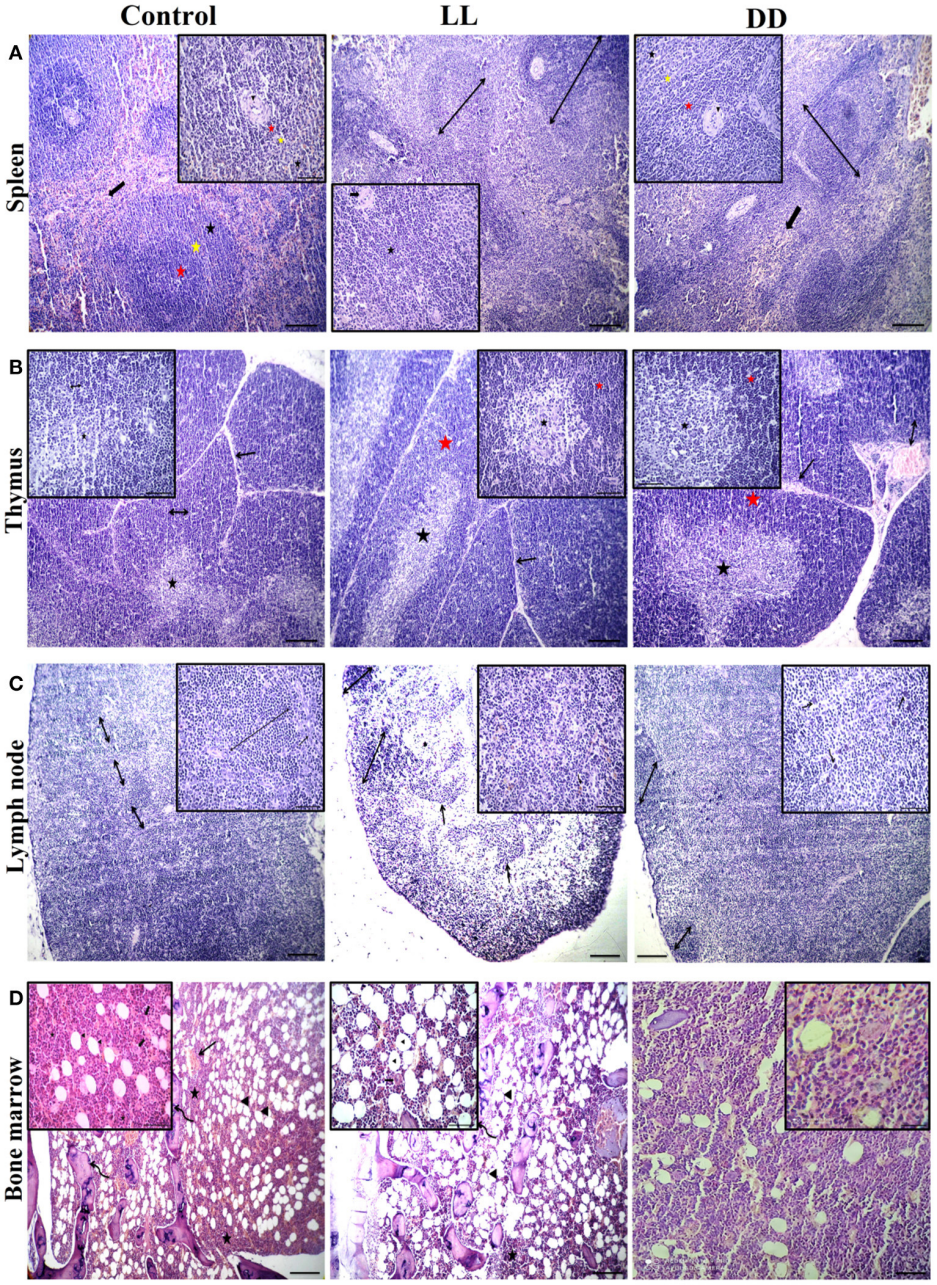
Effect of changing dark/light cycle on lymphoid tissues histology. **(A)** Light micrographs of spleen sections from rats of the control group showing preserved central arteriole (arrowhead), germinal center (red star), mantle zone (yellow star), and marginal zone (black star) of white pulp beside preserved sinusoids (thick arrow) and supporting stroma within the red pulp. The spleen of rats in the LL group revealed moderate splenic activity (star) with enlarged white pulp lymphoid population (double-headed arrows) beside eccentric central arteriole (arrowhead) in addition to dilated sinusoids within red pulp (thick arrow). The spleen of DD rats showing enlarged white pulp lymphoid population (double-headed arrow) beside eccentric central arteriole (arrowhead) with prominent germinal (red star), mantle (yellow star), and marginal zones (black star) in addition to a large number of mature and immature lymphocytes within red pulp (thick arrow) H&E. **(B)** Thymus sections from the control group demonstrating normal cortical (yellow star), and medullary (black star) lymphoid population with normal dividing septa (arrow). Thymus sections from both LL and DD groups showing moderate activity of cortical (yellow arrows) and medullary lymphoid contents (black arrows) with prominent septa (arrow), and dilated septal blood vessels (curved arrow). **(C)** Lymph node sections from the control group showing normal cortical lymphoid follicles (double-headed arrow), medullary lymphatic cords (arrowhead), and trabeculae (arrow). Lymph node sections from the LL group demonstrating cortical lymphoid follicles (double headed arrow heads), lymphatic cords (arrows) and lymphatic sinuses infiltrated with macrophages contain hemosiderin pigment (curved arrows). Lymph node from DD group revealing enlarged cortical lymphatic nodules (double-headed arrows) and infiltrated medulla by proliferated macrophages (curved arrows). **(D)** Bone marrow sections from near epiphysis in the control and LL rats displaying normal cellularity of different hematopoietic series (stars) beside normal megakaryocytic contents (thick arrows), bony spicules of spongy bone (curved arrows), a moderate number of the fat vacuole (arrowheads) and dilated blood sinusoids (arrows). Bone marrow sections from DD rats showing highly active hematopoietic series and a lesser amount of adipocyte. H&E. Scale bar, 50 μm.

The spleen of all examined groups of rats revealed normal splenic histomorphology with preserved white pulp lymphoid arrangement (central arteriole: germinal centers, mantle zone, and marginal zone) and preserved red pulp. The red pulp showed moderate infiltration of mature and immature lymphocytes (Figure 10). The thymus of all groups showed normal cortical and medullary lymphoid population with normal Hassall's corpuscles and septa.

Lymph nodes of the control group revealed normal cortical lymphoid follicles, medullary lymphatic cords, and trabeculae. However, under constant light and constant dark exposure, they showed cortical lymphoid follicles, lymphatic cords, and lymphatic sinuses infiltrated with macrophages containing endogenous pigment (hemosiderin) and small and large lymphocytes beside plasma cells. The bone marrow of the control and continuous light exposure groups showed normal cellularity of different hematopoietic series including myelocytic, lymphocytic, and erythrocytic components besides normal megakaryocytic contents. Bony spicules of spongy bone and a moderate number of fat vacuoles with dilated sinusoids were commonly observed (Figure 10). However, bone marrow sections of the continuous dark exposure group revealed highly active hematopoietic series with a lesser number of adipocytes (Figure 10).

## Discussion

Disruptions of circadian rhythms have been reported to be associated with impaired immune function. Abnormal light exposure affects both the immune system (38–40) and metabolic function (32, 40) of animals. In this study, circulating levels of proinflammatory cytokines (i.e., IL-1β, IL-6, and TNF-α) were significantly decreased by continuous light exposure as well as continuous dark exposure; however, the anti-inflammatory cytokine IL-10 and the immunomodulatory cytokine IL-12 were significantly increased. It has been reported that cytokine production follows circadian rhythmicity, with peak secretion of the proinflammatory cytokines (IL-1β, IL-6, IL-12, and TNF-α) during the night and peak production of anti-inflammatory cytokines (IL-10) during daytime activity (41), to stimulate immediate responses of humoral or specific immunity (42). Furthermore, continuous light exposure heightens the proinflammatory state and induces an increase in the number of neutrophils (43). In the present study, the total number of circulating neutrophils was increased only by constant dark exposure (Table 4). Neutrophils play an important role in the innate defense against pathogens via the secretion of cytokines such as IL-1, IL-6, IL-8, IL-12, IL-10, TNF-α, and IFN-γ (44–46) and proteases (47). Although neutrophils are generally identified as proinflammatory cells, it has been reported that neutrophils are capable of hindering the proinflammatory cytokine production (IL-1β and TNF-a) via protease release, without affecting the anti-inflammatory cytokine IL-10 (48).

Stress and circadian rhythm disruption have reciprocal relationships. Both intersect at the hypothalamic-pituitary adrenal axis and modulate cortisol levels (49). Elevated glucocorticoids in acute and chronic stressful situations reset the circadian clock system (50). Glucocorticoids inhibit proinflammatory cytokines such as IL-1β, IL-6, and TNF-α in a negative feedback mechanism (51). It has been reported that light is capable of inducing a rapid corticosterone response in rats, as in humans (52) and mice (53), which is unlikely to be due to the general activity level of the animal, as light inhibits locomotor behavior in nocturnal animals (54). In the present study, serum corticosterone concentrations were elevated by both continuous light and continuous dark exposure (Figure 2D), which may further explain the decreased levels of proinflammatory cytokines that were demonstrated in this study.

Tumor necrosis factor-alpha regulates various functions of the immune system. One of its functions is the regulation of B-lymphocyte function and immunoglobulin production (55), and its deficiency impairs humoral response, especially the production of IgG (56). Furthermore, hypercortisolemia has adverse effects on lymphocyte-B function and antibody production (57–59). Continuous dark exposure induces significant decreases in the circulating concentrations of IgG, IgM, and IgE antibodies. Moreover, continuous light exposure inhibits IgM and IgE antibody production (Table 5). All of the above indicate a modulatory effect of hypercortisolemia along with decreased TNF-α concentration in diminishing the secretion of different immunoglobulins, which was observed in the present study. However, unexpectedly, IgA was markedly elevated solely by continuous dark exposure. IgA is one of the major antibodies responsible for the humoral mucosal immune system (60). It is well-known that the synthesis of melatonin is higher when it is dark, while light exposure suppresses the activity of the pineal gland. The notion of the existence of a positive correlation between melatonin and IgA (61) supports the present finding of elevation of IgA only by continuous dark exposure.

C-reactive protein is a marker for infection, increases in response to systemic inflammation and is mainly synthesized by hepatocytes in response to proinflammatory cytokines (IL-1, IL-6, and TNF-α) (62). Accordingly, CRP production is a good general indicator for the activity of these cytokines, notably IL-6 (63). In the present study, CRP concentrations were largely increased by continuous light exposure and moderately elevated by continuous dark exposure (Figure 2C) even though levels of the proinflammatory cytokines that mediate the production of CRP were decreased. The exact reason for this is unclear, but it has been reported that abnormal high cortisol levels can disrupt the HPA axis and increase CRP (64). Although the levels of CRP have been demonstrated to be quite stable over 24 h (65), the circadian disruption itself augments the levels of CRP (66).

As reported in the present study, continuous dark exposure showed adverse effects on overall erythrocyte count and its associated parameters, including Hb, PCV, and MHCH (Table 3), except for MCV and MCH, which significantly increased. This indicates a tendency for anemia, which may be as a result of a bone marrow disorder as the histological examination demonstrated hypercellular marrow with all forms of the myeloid cell line being expanded (Figure 10). RBC rhythm amplitudes are very small and physiologically interesting. The number of erythrocytes in the blood is under clock gene control and varies rhythmically (67). The circulating numbers of RBC depend on its production in the bone marrow and Clock mutation has been reported to modulate the diurnal rhythm of bone marrow production of RBC (67). Therefore, the reduction in the number of RBCs noticed in this study may be attributed to hemolysis of red blood cells and/or lower production of RBCs. However, erythrocytes hemolysis can be ruled out because the histological examination of the spleens did not reveal any pathological changes. Lower concentration of Hb therefore may be attributed to the decline in the number of RBCs due to erythropenia and impaired hemoglobin production. Decreased PCV may also be attributed to decreased RBC count coupled with hypohemoglobinemia. However, continuous light exposure induced normocytic hypochromic anemia, as both the MCH and MCHC were significantly decreased, with normal MCV values (Table 3).

The initial response to inflammation is the stimulation of different cells that normally circulate in the blood; leukocytes and thrombocytes (68). Thrombocytes are closely associated with immune systems and control inflammatory responses (69, 70). Both continuous dark and continuous light exposure induced thrombocytopenia (Table 3), which may be due to increased platelet destruction or decreased platelet production as a consequence of elevated corticosterone levels.

The number of leukocytes in the blood displays a daily rhythm (67). Continuous light exposure induced monocytosis, basophilia, and eosinophilia (Table 4), which is most likely attributed to shifting from a marginalization into circulating blood. On the other hand, continuous dark exposure increased the total number of leukocytes, specifically neutrophils, and monocytes (Table 4), suggesting stimulation of cellular immunity mainly via neutrophils and, to a lesser extent, monocytes, which are the first-order immune response. The increased number of leukocytes in peripheral blood may be associated with the phenomenon of cell movement from other lymphoid organs leading to a general increase in the number of blood cells. However, the percentage of lymphocytes was significantly decreased (Table 4), which may be due to lymphocyte homing to lymphoid tissues, especially lymph nodes that peak at night's onset (71). It may also be due to continuous dark exposure incrementing neutrophils that induce inflammation, which in turn decreases lymphocyte count through stress hormone production and thus suppresses the immunological function. In rodents, increased proliferation of lymphocytes in lymph nodes and the spleen during the day has been demonstrated (72), while more lymphocytes have been reported to exist in the thymus and spleen at night (6, 73). In the present study, continuous brightness, as well as continuous darkness, significantly decreased the numbers of CD4^+^ and CD8^+^ T cells as evidenced by the immunohistochemistry of almost all investigated lymphoid organs (Figures 6–9). In mice, the numbers of CD4^+^ and CD8^+^ T cells are increased at the beginning of the dark phase (71). A peak in the number of total T cells and CD4^+^ T cells has been reported during early sleep in different animal and human studies (21, 67), but this rhythm is unclear or absent in CD8^+^ T cells (21, 74).

Circadian rhythm disturbance has negative consequences in various biological systems including immune, inflammatory, and oxidative stress systems (75). Cellular defense against oxidative stress relies on protective enzymes, such as SOD, CAT, and GPx. In animals, sleep deprivation contributes to decreasing the levels of antioxidant enzymes and increasing oxidative stress in the brain (76). Moreover, the CAT level declines and SOD activity elevates after sleep deprivation in rats (77), indicating that an imbalance of antioxidant enzymes occurs after sleep disturbance. Many of these antioxidant enzymes follow circadian rhythms in numerous organisms and tissues (78). Although endogenous melatonin, which is known to be secreted during the night, has been shown to activate the antioxidant enzymes GPx, SOD, and CAT (79), the present results reveal that both continuous light and continuous dark exposure elevates the levels of SOD, CAT, GPx and TAC in the spleen, lymph node and bone marrow (Figures 4, 5, respectively). However, in the thymus, continuous light exposure significantly decreased SOD, CAT, GPx, and TAC while continuous dark exposure significantly increased TAC and decreased GPx, suggesting that the response to circadian disruption is tissue specific. The expression levels of SOD have been demonstrated to oscillate with a daily rhythm in rat intestine, lung, and cerebellum (80). In nocturnal mice, the activity of CAT enzymes has been shown to peak in the middle of the dark phase in the liver and kidneys (81). However, the peak occurs at the beginning of the light phase in diurnal humans (82). Continuous light exposure diminishes the circadian rhythms of SOD and CAT that are observed under normal light and dark in Syrian hamsters (83). The circadian variation of GPx enzymes was also reported (79, 84). In addition, GPx acrophase has been demonstrated to peak in the phase opposite to that of lipid peroxidation, which is elevated during the dark phase (85). This suggests that the peak in lipid peroxidation is partly due to the low levels of protective enzymes at night (86). The lipid peroxidation marker MDA is produced by free radicals, and melatonin has been demonstrated to prevent lipid peroxidation in different tissues (87). In the present study, both antioxidant defense mechanisms and lipid peroxidation were significantly increased in the spleen and axillary lymph node following continuous light or dark exposure (Figures 4, 5, respectively) suggesting a positive correlation. A possible explanation is that continuous light and dark exposure increased lipid peroxidation and subsequent oxidative stress in such tissues, which in turn stimulated compensatory changes in the levels of some antioxidants such as SOD, CAT, and GPx. These changes consecutively provide further protection against lipid peroxidation.

In addition to the well-known classical stressors, circadian disruption *per se* can be considered a stressor, since it can change glucocorticoid release. During stress, NO production is exaggerated and triggers the lipid peroxidation reactions, possibly due to its conversion to per-oxy-nitrite radical (88). NO-dependent signaling is engaged in circadian rhythms. NO participates in adapting the system to the conditions of environmental lighting and production of NO connect retinal light activation to cellular changes within the SCN which mediate the biological clock reset process (89–91). Therefore, in this study, NO in part may contribute to the elevated levels of lipid peroxidation. The protective efficiency against lipid peroxidation depends on the balance between ROS and the availability of antioxidant defenses. This was clear in the bone marrow, whereas the elevated antioxidant enzyme levels were sufficient to suppress lipid peroxidation biomarker MDA. On the contrary, in the thymus, continuous light exposure diminished the levels of antioxidant enzymes along with the levels of lipid peroxidation (Figures 4, 5), suggesting overutilization of the antioxidant enzymes to scavenge the lipid peroxidation products in the light-exposed group. It has been reported that constant light elevates MDA levels in different tissues (92).

Several peripheral tissues have autonomous circadian clocks such as liver, kidney, pancreas, ovary, testes, lung, and skeletal muscles (93–95), which are important regulators of normal peripheral physiology. To understand the role of the circadian clock in modulating immune function, the expression of core clock genes in the spleen was investigated, and the results show upregulation in Cry1, Cry2, Per1, Per2, and Per3 mRNA by continuous dark exposure. However, Cry1 and Cry2 mRNA expression was downregulated by continuous light exposure (Figure 3), indicating the existence of a functional circadian clock in the spleen. It has been demonstrated that Cry1 is quickly and powerfully induced in pars tuberalis by melatonin administration and peaks in the dark phase when melatonin exists in the bloodstream (96). However, Per1 mRNA peaks early in the day, when blood melatonin is low (96). Per2 mRNA peaks in macrophages just after the animal enters the active phase (6). Moreover, in peripheral tissues, Per1 and Per2 genes in natural killer cells are expressed in antiphase, peaking during the day and night, respectively (97).

In conclusion, the present study examined different immune responses in rats exposed to continuous brightness or darkness or normal light/dark cycle and found that chronic circadian disruption influences corticosterone levels, have adverse effects on hematological parameters, dampens inflammatory responses, increases antioxidant enzyme activities, and modifies core clock genes expression in rat spleen.

## Data Availability Statement

The datasets presented in this study can be found in online repositories. The names of the repository/repositories and accession number(s) can be found below: https://www.ncbi.nlm.nih.gov/genbank/, NM_198750.2; NM_133405.2; NM_0010341; NM_031678.125.1; NM_023978.2; NM_031144.

## Ethics Statement

The animal study was reviewed and approved by the Ethics Committee of the Faculty of Veterinary Medicine, Zagazig University (ZU-IACUC/2/F/139/2019).

## Author Contributions

AM contributed to the design and implementation of the research, to the analysis of the results, and to the writing of the manuscript.

## Conflict of Interest

The author declares that the research was conducted in the absence of any commercial or financial relationships that could be construed as a potential conflict of interest.
